# Plasma IL-8 and ICOSLG as prognostic biomarkers in glioblastoma

**DOI:** 10.1093/noajnl/vdab072

**Published:** 2021-06-01

**Authors:** Camilla Bjørnbak Holst, Ib Jarle Christensen, Kristoffer Vitting-Seerup, Jane Skjøth-Rasmussen, Petra Hamerlik, Hans Skovgaard Poulsen, Julia Sidenius Johansen

**Affiliations:** 1 Department of Radiation Biology, Department of Oncology, Rigshospitalet, Copenhagen University Hospital, Copenhagen, Denmark; 2 Brain Tumor Biology, Danish Cancer Society Research Center, Danish Cancer Society, Copenhagen, Denmark; 3 Department of Oncology, Herlev and Gentofte Hospital, Copenhagen University Hospital, Herlev, Denmark; 4 Department of Medicine, Herlev and Gentofte Hospital, Copenhagen University Hospital, Herlev, Denmark; 5 Department of Clinical Medicine, Faculty of Health and Medical Sciences, University of Copenhagen, Copenhagen, Denmark; 6 Department of Gastroenterology, Hvidovre Hospital, Hvidovre, Denmark; 7 Bioinformatics Centre, Department of Biology, Biotech Research and Innovation Centre (BRIC), University of Copenhagen, Copenhagen, Denmark; 8 Department of Neurosurgery, Rigshospitalet, Copenhagen University Hospital, Copenhagen, Denmark

**Keywords:** circulating biomarkers, glioblastoma, ICOS ligand, IL-8

## Abstract

**Background:**

CNS immune privilege has been challenged in recent years. Glioblastoma (GBM) immune dysfunction includes complex interactions with the immune system outside the CNS. The aim of this study was to determine diagnostic and prognostic potential of immune-related proteins in plasma in GBM and interrogate biomarker presence in the brain tumor microenvironment (TME).

**Methods:**

One hundred and fifty-eight patients with glioma WHO grade II–IV were included. Plasma collected at surgery was screened for 92 proteins using proximity extension assay technology and related to clinical outcome. Secretion and expression of candidate prognostic biomarkers were subsequently analyzed in 8 GBM cell lines and public RNAseq data.

**Results:**

Plasma levels of 20 out of 92 screened proteins were significantly different in patients with GBM compared to patients with astrocytoma WHO grade II–III. High plasma interleukin-8 (IL-8) (hazard ratio [HR] = 1.52; *P* = .0077) and low CD244 (HR = 0.36; *P* = .0004) were associated with short progression-free survival and high plasma IL-8 (HR = 1.40; *P* = .044) and low ICOS ligand (ICOSLG) (HR = 0.17; *P* = .0003) were associated with short overall survival (OS) in newly diagnosed patients with GBM. A similar trend was found for ICOSLG (HR = 0.34; *P* = .053) in recurrent GBM. IL-8 was mostly secreted and expressed by mesenchymal GBM cell lines and expressed by vascular cells and immune cells in the TME. This was also the case for ICOSLG, although less consistent, and with additional expression in tumor-associated oligodendrocytes.

**Conclusions:**

High plasma IL-8 and low ICOSLG at surgery are associated with short OS in newly diagnosed GBM. Source of plasma ICOSLG may be found outside the TME.

**Key Points**

- High plasma IL-8 at surgery is associated with short overall survival in glioblastoma.
- Low plasma ICOS ligand at surgery is associated with short overall survival in glioblastoma.

**Importance of the Study**

The brain tumor microenvironment (TME) is undeniably unique with resident cell types and interactions contributing to immune dysfunction in glioblastoma (GBM). However, the paradigm shift defying CNS immune privilege has made systemic immune status an important stakeholder in assessment of GBM immune modulation. We investigated prognostic and diagnostic potential of 92 immune-related proteins in plasma from patients with glioma. High plasma interleukin-8 (IL-8) and low ICOS ligand (ICOSLG) were associated with short overall survival (OS) in patients with newly diagnosed GBM. For ICOSLG, a similar trend was found in patients with recurrent GBM. The chemokine IL-8 promotes tumor propagation and angiogenesis in GBM consistent with its negative prognostic impact. ICOSLG is a membrane-associated checkpoint molecule and is described as tumorigenic in the TME. Association of low plasma ICOSLG with short OS emphasize that the systemic immune response and soluble membrane-associated proteins may have independent functions in GBM.

Immunotherapy has revolutionized treatment of many types of cancers like melanoma, lung, and kidney cancer.^1^ Glioblastoma (GBM) does not appear on this list^2,3^ and survival has practically remained unchanged for 2 decades.^4^ GBM growth and treatment resistance are nurtured by its heterogeneous, dynamic, infiltrative nature, a unique brain tumor microenvironment (TME), low mutational burden, and immune dysfunction.^2^ At present, GBM diagnosis, response, and prognosis are assessed through clinical observations, tissue biopsies, and neuroimaging.^5^ The paradigm describing the brain as an immune-privileged site lacking lymphatic drainage has changed with the acknowledgment of significant interactions between the brain and the immune system.^2,3,6^ In addition, GBM is characterized by a partially compromised blood–brain barrier leaking tumor-associated proteins.^7^ The simple noninvasive analysis of circulating protein biomarkers therefore presents as an intriguing approach to monitor glioma development. Recent advances in proteomic technology enable high-throughput multiplex screening and therefore the possibility to assess complex interactions and screen for prognostic and predictive biomarkers. To evaluate systemic immune dysfunction in GBM, we screened 92 immune-related proteins in plasma from 158 patients with glioma WHO grade II–IV to assess diagnostic and prognostic potential. Candidate prognostic biomarkers were further interrogated in 8 paired GBM cell lines and publicly available RNA sequencing datasets.

## Materials and Methods

### Patients and Patient Samples

We retrospectively included 158 patients with histologically confirmed WHO grade II–IV gliomas (Cohort 1) and this cohort has been described previously.^8^ Plasma samples from the patients were available from the Copenhagen Brain Tumor Consortium (CBTC) Glio Research Biobank. Sample size was limited by availability of plasma samples. Perioperative venous blood samples were collected in EDTA vials (VACUETTE K2E K2EDTA) and stored at 4°C/on ice for a maximum of 2 h. Plasma was aliquoted after centrifugation at 3000 rpm for 10 min at 4°C and stored at −80°C until analysis.

Patients with GBM (*n* = 134) from the main study cohort (Cohort 1) were further divided into 2 study populations: Cohort 2 including 94 patients with GBM and blood samples from initial surgery and Cohort 3 including 40 patients with GBM and blood samples from relapse surgery. Paired plasma samples from relapse surgery were available for 11 patients (Cohort 4) in Cohort 2.

The study protocol was approved by the Danish Regional Committee on Health Research Ethics (H-3-2009-136). Informed written consent was obtained from all participants.

Tumor and patient characteristics were obtained retrospectively through review of medical charts, pathology reports and MRI descriptions. Patients were followed until death or end of follow-up (May 14, 2018). Date of progression was found through patient charts and MRI descriptions and based on clinician’s assessment.

### Cell Lines

GBM cell cultures derived from tumor tissue from patients in Cohort 1 have recently been described and characterized^9^ and were maintained through subcutaneous implantation in the flanks of immunocompromised NOD.Cg-Prkdc^scid^ mice (Taconic Biosciences, Inc., cat. no. NOG-F). The protocol was approved by the Danish Regulations for Animal Welfare (Protocol Number 2012-15-2934-00636/2018-15-0201-01391). GBM cells were cultured in Neurobasal A media (Invitrogen, cat. no. 12349-015) supplemented with B27 supplement minus vitamin A (Invitrogen, cat. no. 12587-010), epidermal growth factor (20 ng/mL; Invitrogen, cat. no. PHG0313), and basic fibroblast growth factor (20 ng/mL; Invitrogen, cat. no. PHG0263), Glutamax (Invitrogen, cat. no. 35050-038) and penicillin/streptomycin (Invitrogen, cat. no. 15140-122) (COMP). For a limited time period cell cultures were supplemented with up to 1% fetal bovine serum (FBS) (Invitrogen, cat. no. 26140095) to facilitate growth. Experiments were performed in COMP without FBS. Cells were cultured at 37°C in an atmosphere of 5% CO_2_. Prior to experiments, single-cell suspensions were prepared using TrypLE (Invitrogen, cat. no. 12563011) followed by Trypan Blue Stain (Invitrogen, cat. no. T20393) to exclude dead cells before counting viable cells on the Countess II Automated Cell Counter (Thermo Fisher Scientific).

#### Conditioned media

Cells were plated at 4 × 10^5^ viable cells/mL COMP in triplicates and conditioned medium was collected after 72 h following centrifugation at 2000 rpm for 5 min at 4°C. If cell pellets were collected samples were initially centrifugated at 1200 rpm for 5 min at 4°C.

#### RNA sequencing

Total RNA from GBM cell cultures was isolated using the AllPrep DNA/RNA/Protein mini kit (80004, Qiagen) according to the manufacturer’s instructions. Purified RNA was sent for 100 nt paired end strand specific “BGI-Seq LncRNA-seq(mRNA+lncRNA)” sequencing (obtained via rRNA depletion) at BGI China. The resulting sequences (FASTQ files) were quality controlled using FastQC (http://www.bioinformatics.babraham.ac.uk/projects/fastqc/) and used to quantify GENCODE v26 transcripts^10^ using Salmon v 0.13.1^11^ with the parameters “-seqBias,” “--gcBias,” and “--validateMappings.” IsoformSwitchAnalyzeR^12^ was used to import the data into R, perform interlibrary normalization of the TPM values, and summarize to gene expression levels.

### Protein Screening Using Proximity Extension Assay

Proximity extension assay (PEA) was performed by BioXpedia A/S using the Olink Immuno-Oncology panel (www.olink.com). Plasma samples and conditioned media from GBM cell lines were thawed at 4°C, vortexed, and centrifuged at 400g for 1 min. One µL of plasma was added to 3 µL incubation mix in a 96-well plate, with each well containing 92 pairs of oligonucleotide conjugated antibodies, incubation solution, and incubation stabilizer. Negative controls, interplate controls, and control samples were added to the plate. Following overnight incubation at 4°C 96 µL extension mix (PEA solution, PEA enzyme, and PCR polymerase) was added to each well. The plate was vortexed, centrifuged, and transferred to a thermal cycler (Veriti 96 well Thermal Cycler, Applied Biosystems) for initial DNA extension at 50°C for 20 min followed by 17 cycles of DNA amplification. DNA amplicons for each protein were subsequently quantified using the Fluidigm Biomark system (Fluidigm).

#### PEA quantification

The relative quantification of protein abundance was measured in Normalized Protein Expression (NPX), which is an arbitrary unit on log2 scale. High NPX corresponds to high protein abundance. NPX values were calculated from cycle threshold values (Ct values) exported from the Fluidigm Real-Time PCR Analysis software (Fluidigm) using NPX Manager software (Olink Proteomics). Within the lower and upper limit of quantification a 1-unit increase in NPX corresponds to a 2-fold increase in protein abundance. Using NPX Manager the Ct values were normalized against extension and interplate controls and a correction factor. NPX values for all plates were normalized using intensity normalization. Assay specific limit of detection (LOD) was calculated as 3 times the standard deviation over the background signal.

Plasma IL-6 has previously been measured by ELISA in the same study cohort.^8^ Comparing plasma IL-6 measurements performed with a well-described commercial ELISA (Quantikine high sensitive IL-6 catalogue HS600, R&D Systems) and the PEA technology (Olink) revealed comparable results (moment correlation: *r* = 0.92) serving as additional control of the novel PEA technology.

Four samples deviated significantly from quality control criteria. Three of these samples were paired samples from the same patient. We did not exclude any samples from further analysis.

Out of 182 samples (Cohort 1: 158; Cohort 4: 11; Controls: 8; Not relevant: 5) IL-1α analysis failed for 8 samples and MCP-3 failed for 58 samples (including 2 controls). MCP-3, IL-1α, IFN-β, IL-2, IL-33, CD28, IL-35, IL-5, IFN-γ, IL-21, IL-13, and TNF all had above 80% of values below LOD (or missing for MCP-3 and IL-1α). The complete biomarker panel, LOD and percentage of data below LOD/missing are presented in Supplementary Table S1.

### Endpoints

Progression-free survival (PFS) was defined as time from initial GBM diagnosis (same date as blood sampling) until first relapse with radiological or clinical progression or death without prior history of relapse. Overall survival (OS) was calculated as time from blood sampling until date of death of any cause or end of follow-up (May 14, 2018). Two patients in Cohort 2 (newly diagnosed GBM) were lost to follow-up after first recurrence and were censored for OS analysis.

### RNA Sequencing of Purified Cells From Healthy Juvenile/Adult Brain, Fetal Astrocytes and Astrocytes From Diseased Brain

Zhang et al. purified specific cell populations from human brain samples through immunopanning.^13^ Anti-CD45 antibody (microglia/macrophages), anti-GalC hybridoma (oligodendrocytes and myelin debris), anti-O4 hybridoma (oligodendrocyte precursor cells), anti-Thy1 antibody (neurons), anti-HepaCAM antibody (astrocytes), and Bandeiraea simplicifolia lectin 1 (BSL-1) (endothelial cells) were used to bind the different cell types (further details regarding RNAseq data can be found in Zhang et al.).^13^ Astrocytes from GBM core were taken from regions with contrast enhancement. RNAseq quantification in FPKM (fragments per kilobase of transcript sequence per million mapped fragments) was generously shared by the Barres Lab (Dr. Steven Sloan).

### Single-Cell RNA Sequencing of the GBM Microenvironment

The single-cell RNAseq quantifications from Darmanis et al.^14^ were downloaded from www.GBMseq.org along with the cell types. The marker gene expression was evaluated by calculating the mean of the nonzero cells. For each gene these values were zero mean unit variance scaled.

### Immune Cell Fractions and Biomarker Analysis in the TCGA Dataset

TCGA^15^ primary GBM microarray expression was obtained from GlioVis.^16^ Immune cell type fractions were obtained from Wang et al. using their Supplementary Table S5.^17^ Correlation analysis was done in R with cor.test using the Spearman method.

### TCGA Survival Analysis

TCGA LGG and GBM microarray data and associated survival information were obtained from GlioVis.^16^ Expression data were log2 transformed with a pseudocount of 1 and afterwards mean-variance scaled. Patient age was obtained from TCGA CDR.^18^ Survival analysis was performed using multivariate cox proportional hazards regression as implemented in the “coxph” function from the “survival” R package.^19^ For each individual, as well as the combined dataset, we created cox regression which analyzed the OS hazard ratio (HR) as a function of the tumor grade, isocitrate dehydrogenase (IDH) status, MGMT methylation status (in GBM), age, gender, and expression data for the IL-8 and ICOSLG genes.

### Statistical Analysis

A general linear model (GLM) was used to evaluate association between plasma protein levels and categorical variables (MGMT status, age, sex, and treatment). Spearman rank correlations were used to assess correlation between selected plasma protein levels and corticosteroid dosage. Comparison of astrocytoma WHO grade II–III and GBM or oligodendroglioma WHO grade II–III with the screened proteins as explanatory variables were done using a GLM adjusting for age and sex and presenting *P* for the difference.

In Cohort 2 (newly diagnosed GBM) associations between plasma protein levels and PFS or OS, respectively, were tested using the Cox proportional hazards model estimating univariate and multivariate-adjusted HRs and 95% confidence intervals (CIs). The proportional hazards assumption and linearity were evaluated with martingale residuals. For the 92 proteins assayed in the Olink analysis, univariate analysis of each protein was performed for PFS and OS in Cohort 2 and a panel of candidates for further investigation was selected choosing those with *P* less than 5%. These panels representing PFS and OS were then included in a multivariate analysis with backwards stepwise selection and 10-fold cross-validation choosing only markers significant at the 1% level.^20^ The proteins identified for each outcome were then included in multivariate analysis with the addition of clinical variables age, sex, MGMT promotor methylation, and treatment regimen. In the final model *P* less than 5% was considered significant. The model developed for Cohort 2 (newly diagnosed GBM) was then locked and applied directly to Cohort 3 (recurrent GBM) with no fitting.

Calculations and graphs were made using SPSS (v22.0, IBM Corp.), GraphPad Prism (v8.0.0, GraphPad Software), SAS (v9.4), and R version 3.6.3 (2020-02-29).

## Results

### Patient Characteristics

Patient characteristics are presented in Supplementary Tables S2–S4. Information on corticosteroid consumption at time of blood sampling was available for 82 out or 94 patients with newly diagnosed GBM. Only 2 patients with newly diagnosed GBM did not receive corticosteroid treatment at surgery. Three patients received immunotherapy at some point during their disease course. These 3 patients were all part of Cohort 3 (recurrent GBM). All oligodendrogliomas WHO grade II–III with known IDH status (6 out of 7) and 13 out of 17 astrocytomas WHO grade II–III presented with an IDH mutation, whereas 3 patients (out of 131 with available IDH status) with GBM had IDH mutation according to pathology reports from previous, baseline, or later surgeries.

### Plasma Immune Profiles Differ Between Tumor Grades

Twenty out of 92 screened proteins in plasma showed differential abundance between astrocytoma WHO grade II–III and GBM, when age and sex were included in the model (Table 1). Plasma levels of 4 proteins were significantly different between astrocytoma and oligodendroglioma WHO grade II–III (Table 1).

**Table 1.** Distribution of Proteins in Plasma From Patients With Glioma WHO Grade II–IV

Protein	Dir	Astrocytoma WHO Grade II–III vs GBM	Dir	Astrocytoma WHO Grade II–III vs Oligodendroglioma WHO Grade II–III
		*P* ^a^		*P* ^a^
Caspase-8 (CASP-8)	—	.14	**U**	**.0049**
C-C motif chemokine 4 (CCL4)	**U**	**.025**	—	.19
C-C motif chemokine 19 (CCL19)	**U**	**.0051**	—	.80
T-cell surface glycoprotein CD5 (CD5)	**U**	**.0010**	—	.76
CD70 antigen (CD70)	**U**	**.0050**	—	.76
CD83 antigen (CD83)	**U**	**.0048**	—	.60
Natural killer receptor 2B4 (CD244)	**U**	**.026**	—	.053
CD 40 ligand (CD40-L)	—	.10	**U**	**.029**
C-X-C motif chemokine 13 (CXCL13)	**D**	**.023**	—	.68
Pro-epidermal growth factor (EGF)	—	.24	**U**	**.022**
Tumor necrosis factor ligand superfamily member 6 (FASLG)	**U**	**.041**	—	.24
Granzyme A (GZMA)	**U**	**.025**	—	.97
Granzyme B (GZMB)	**U**	**.0043**	—	.13
Galectin-1 (Gal-1)	**U**	**.036**	—	.26
Interleukin-12 (IL-12)	**U**	**.0005**	—	.91
Interleukin-1 alpha (IL-1α)	—	.92	**D**	**.0025**
C-C motif chemokine 8 (MCP-2)	**U**	**.014**	—	.18
Matrix metalloproteinase-12 (MMP-12)	**U**	**.0026**	—	.97
Programmed cell death protein 1 (PDCD1)	**U**	**.0040**	—	.65
Programmed cell death 1 ligand 2 (PD-L2)	**U**	**.042**	—	.78
Pleiotrophin (PTN)	**U**	**.0014**	—	.29
Tumor necrosis factor receptor superfamily member 9 (TNFRSF9)	**U**	**.032**	—	.87
Tumor necrosis factor receptor superfamily member 21 (TNFRSF21)	**U**	**.015**	—	.56
Tumor necrosis factor ligand superfamily member 12 (TWEAK)	**U**	**.0002**	—	.097

Age and sex are included in the analysis. Bold values represent P < .05. D, downregulated; Dir, direction; U, upregulated.

^a^General linear model.

### Plasma Immune Profiles Associated With Survival in Newly Diagnosed GBM

In univariate analysis 16 out of 92 proteins were associated with PFS and 14 proteins were associated with OS (Supplementary Table S5) in 94 patients with newly diagnosed GBM. High protein levels were most often associated with long PFS and/or OS, except for interleukin-8 (IL-8) (PFS: HR = 1.56, 95% CI: 1.18–2.07; OS: HR = 1.41, 95% CI: 1.06–1.88) and Pleiotrophin (OS: HR = 1.24, 95% CI: 1.03–1.49) where high protein levels were associated with short PFS and/or OS.

In multivariate analysis with cross-validation and inclusion of clinical variables, high plasma natural killer receptor 2B4 (CD244) was associated with long PFS and high IL-8 was associated with short PFS. In multivariate analysis with cross-validation and inclusion of clinical variables high ICOSLG (ICOS ligand) (gene name: *ICOSLG*) was associated with long OS and high IL-8 (gene name: *CXCL8*) was associated with short OS (Table 2). Plasma IL-8 was associated with treatment regimen and age, whereas plasma ICOSLG was 9% lower in females compared to males (Table 3). Neither IL-8 (*r* = 0.18, *P* = .12) nor ICOSLG (*r* = −0.13, *P* = .23) were correlated with corticosteroid dosage in the 82 patients with newly diagnosed GBM and available information on corticosteroid intake. However, only 2 patients did not receive corticosteroid treatment at time of diagnosis (Supplementary Table S3). Due to the low occurrence of IDH mutations in Cohort 2, we did not include this in multivariate analysis. When we removed the 3 patients with IDH mutated GBM from multivariate analysis, all included factors in the final model (Table 2) remained significantly (*P* < .05) associated with survival.

**Table 2.** Immune-Related Proteins in Plasma Associated With Prognosis in Patients With Newly Diagnosed GBM—Multivariate Analysis Including Clinical Variables

Multivariate Analysis	PFS (*n* = 90)	
	HR (95% CI)	*P* ^a^
Multivariate Analysis	OS (*n* = 91)	
	HR (95% CI)	*P* ^a^
Treatment Stupp vs None/RT/TMZ^b^	0.19 (0.09–0.39)	**<.0001**
MGMT Met vs Non-met	0.46 (0.28–0.74)	**.0014**
Age (years) Per 10 years	0.99 (0.76–1.29)	.96
Sex F vs M	1.16 (0.74–1.82)	.53
Natural killer receptor 2B4 (CD244)	0.36 (0.20–0.63)	**.0004**
Interleukin-8 (IL-8)	1.52 (1.12–2.07)	**.0077**
Treatment Stupp vs None/RT/TMZ^b^	0.29 (0.14–0.57)	**.0004**
MGMT Met vs Non-met	0.42 (0.25–0.71)	**.0010**
Age (years) Per 10 years	1.09 (0.81–1.45)	.57
Sex F vs M	1.52 (0.93–2.48)	.094
ICOS ligand (ICOSLG)	0.17 (0.065–0.45)	**.0003**
Interleukin-8 (IL-8)	1.40 (1.01–1.95)	**.044**

Bold values represent P < .05. CI, confidence interval; F, female; GBM, glioblastoma; HR, hazard ratio; M, male; Met, methylated; Non-met, non-methylated; OS, overall survival; PFS, progression-free survival; RT, radiotherapy; TMZ, temozolomide.

^a^Cox regression analysis.

^b^Treatment regimens were dichotomized into no treatment, radiotherapy only and temozolomide only versus Stupp’s regimen (radiotherapy, concomitant and adjuvant temozolomide) and radiotherapy with concomitant temozolomide.

**Table 3.** Plasma IL-8 and ICOSLG and Patient Characteristics for Patients With Newly Diagnosed GBM

Plasma Biomarkers and Patient Characteristics	Plasma IL-8			Plasma ICOSLG		
	Ratio^a^	95% CI	*P* ^b^	Ratio^a^	95% CI	*P* ^b^
MGMT Met vs Non-met	1.07	0.85–1.36	.55	0.92	0.85–1.01	.065
Age (years) Per 10 years	1.16	1.05–1.29	**.0038**	0.97	0.94–1.01	.18
Sex F vs M	0.92	0.73–1.16	.50	0.91	0.83–0.98	**.019**
Treatment None/RT/TMZ vs Stupp^c^	1.44	1.13–1.83	**.0035**	0.93	0.85–1.02	.10

Bold values represent P < .05. CI, confidence interval; F, female; GBM, glioblastoma; ICOSLG, ICOS ligand; IL-8, interleukin-8; M, male; Met, methylated; Non-met, non-methylated; RT, radiotherapy; TMZ, temozolomide.

^a^Ratio of plasma biomarker level between categories for each variable.

^b^A general linear model was used to evaluate association between plasma protein levels and categorical variables.

^c^Treatment regimens were dichotomized into no treatment, radiotherapy only and temozolomide only versus Stupp’s regimen (radiotherapy, concomitant and adjuvant temozolomide) and radiotherapy with concomitant temozolomide.

### ICOSLG and IL-8 in Recurrent GBM

The prognostic model for OS found in patients with newly diagnosed GBM using plasma ICOSLG and IL-8 was not significantly associated with OS (*P* = .12) in the cohort of 40 patients (Cohort 3) with a diagnosis of recurrent GBM at time of blood sampling. When analyzing plasma IL-8 and ICOSLG individually, high ICOSLG was borderline associated with long OS (HR = 0.34; *P* = .053), whereas IL-8 was not associated with OS (HR = 0.94; *P* = .84).

### Changes in Plasma Immune Profiles Over Time

**Figure 1.**
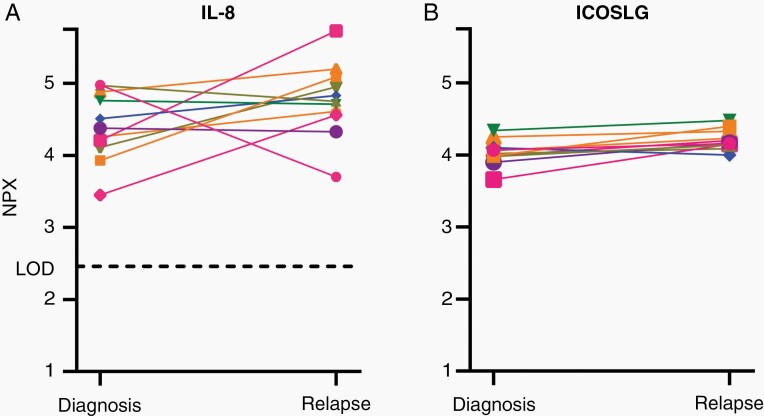
Paired plasma IL-8 (A) and ICOSLG (B) values from initial GBM surgery and recurrence (*n* = 11). One-unit increase in NPX corresponds to a 2-fold increase in protein abundance. GBM, glioblastoma; ICOSLG, ICOS ligand; IL-8, interleukin-8; LOD, limit of detection; NPX, Normalized Protein Expression.

Paired plasma samples from diagnosis and relapse surgery were available for 11 patients with GBM. Analyzing IL-8 and ICOSLG, 3 patients doubled IL-8 and 1 patient had a reduction in IL-8 of more than 50% (Figure 1A). ICOSLG revealed only little variation over time and between patients (Figure 1B).

### Correlation Between Plasma Immune Profiles and the Brain TME

**Figure 2.**
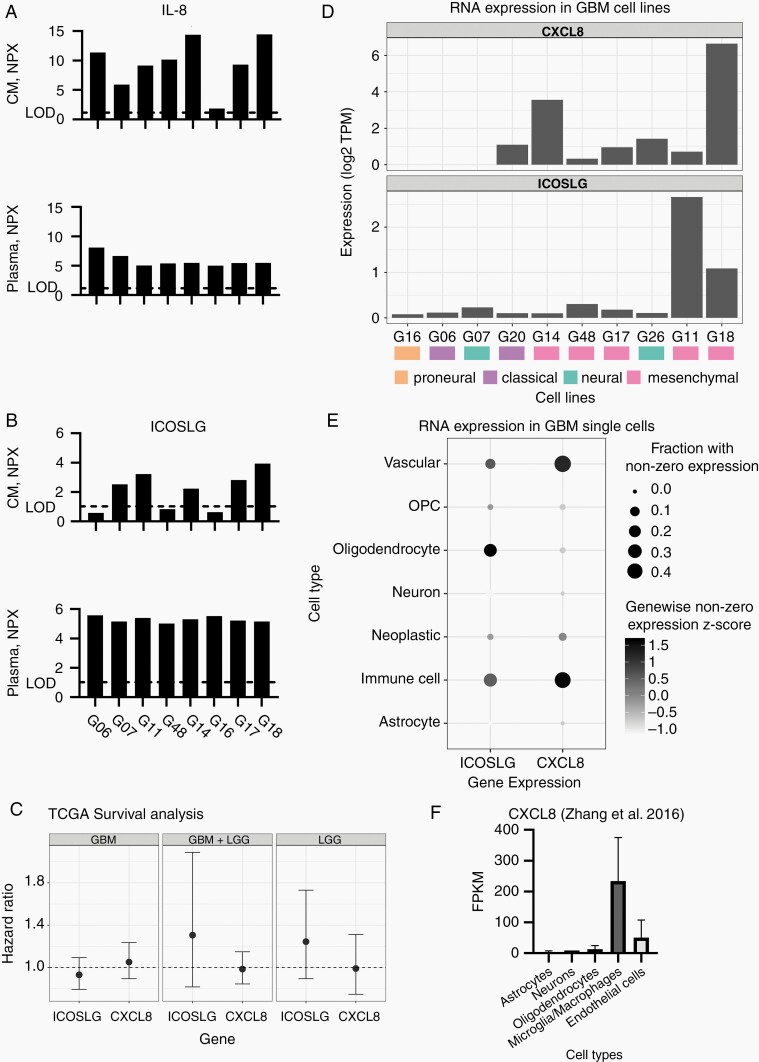
IL-8 and ICOSLG levels in patient plasma and cell line conditioned media from 8 patients and paired cell lines are shown in (A) and (B). In (C) association between *CXCL8*, *ICOSLG* RNA expression and OS in TCGA LGG and GBM datasets are illustrated using Cox proportional hazards regression. Cell line *CXCL8* and *ICOSLG* RNA expression in (D). (E) depicts RNA expression of *CXCL8* and *ICOSLG* in GBM single cells^14^ and (F) depicts RNA expression of *CXCL8* in purified cells from human brain.^13^ In (F) astrocytes include mature astrocytes, fetal astrocytes, and astrocytes from sclerotic hippocampi and GBM. One-unit increase in NPX corresponds to a 2-fold increase in protein abundance. CM, conditioned media; CXCL8, C-X-C Motif Chemokine Ligand 8; GBM, glioblastoma; ICOSLG, ICOS ligand; IL-8, interleukin-8; LGG, low-grade glioma; LOD, limit of detection; NPX, Normalized Protein Expression; OS, overall survival.

We then compared plasma levels of IL-8 and ICOSLG from 8 patients with immune profiles in conditioned media and RNA sequencing from paired GBM cell lines to investigate whether there was a correlation between plasma protein levels and tumor cell expression and secretion. For 6 out of 8 patients IL-8 was higher in conditioned media from tumor cell lines than in plasma (Figure 2A). All mesenchymal cell lines (*n* = 5) based on Verhaak molecular classification^21^ presented with high IL-8 secretion and measurable *CXCL8* RNA expression. Five cell lines secreted ICOSLG (>LOD), but with NPX values lower than for paired patient plasma samples (Figure 2B). Cell line secretion and RNA expression did not fully overlap (Figure 2D). One cell line expressed (Figure 2D) and secreted (Figure 2A and B) minimal/no IL-8 or ICOSLG. This was not reflected in lower plasma values but characterized the only proneural cell line.

Exploring the TCGA dataset, *CXCL8* and *ICOSLG* RNA expression in GBM and low-grade gliomas were not associated with OS (Figure 2C).

To evaluate whether *CXCL8* and *ICOSLG* were expressed by other cells than tumor cells in the brain TME, we interrogated previously published RNA sequencing data from purified cerebral cell populations^13^ and single-cell RNA data from GBM specimens.^14^ In healthy human brain and GBM *CXCL8* was mainly expressed in immune cells and vascular cells (Figure 2E and F). *ICOSLG* was not present in cell populations extracted from healthy adult brain (data not shown) but to some extent in immune cells, vascular cells, and oligodendrocytes in the brain TME (Figure 2E).

To estimate association of local protein expression with immune cell composition in the TME we correlated *CXCL8* and *ICOSLG* expression in TCGA GBM data with matched immune cell type fractions estimated by Wang et al.^17^ Expression of *CXCL8* and *ICOSLG* in the TCGA dataset revealed significant positive correlation with neutrophils, resting NK cells and activated CD4 memory T cells and negative correlation with resting CD4 memory T cells, resting mast cells, activated NK cells, and follicular helper T cells. *ICOSLG* was also negatively correlated with activated dendritic cells and positively correlated with the presence of M2 macrophages, whereas *CXCL8* was negatively correlated with M2 and M1 macrophages and positively correlated with M0 macrophages and activated mast cells (Supplementary Table S6 and Figure S1).

## Discussion

With the advent of increasingly sensitive technologies detecting cell-free DNA, circulating tumor cells, and multiplex panels of proteins in blood samples, a small amount of blood may prove instrumental to GBM diagnosis and treatment strategy in the future. These tools are urgently needed considering the multitude of failed clinical trials, lack of predictive and prognostic biomarkers, intra- and intertumoral heterogeneity and difficulties in sample collection from brain tumors.

We screened 92 immune-related proteins in plasma from patients with newly diagnosed and recurrent GBM and lower grade gliomas using PEA technology. For 12 of these proteins, more than 80% of samples presented with an NPX value below LOD (or missing for MCP-3 and IL-1α). We suspect that this mainly reflects inadequate assay sensitivity for some proteins whereas others are probably not present in plasma.

Twenty proteins showed significant differences in abundance between astrocytoma WHO grade II–III and GBM, indicating that systemic immune status differs between tumor grades. However, we cannot exclude that differences in IDH mutation status and corticosteroid consumption, that have been found to associate with immune phenotype,^22,23^ may contribute to herein observed differences. In newly diagnosed GBM, we found high plasma IL-8 and low plasma ICOSLG at surgery to be associated with short OS in multivariate analysis.

In GBM, IL-8 has been associated with proliferation, invasion, angiogenesis, and vascular mimicry.^24,25^ Few studies have previously evaluated the prognostic impact of plasma IL-8 and did not find prognostic significance,^26,27^ but these results are based on smaller cohorts (14–23 patients) of GBM patients, which may be too few patients to reveal a potential association with survival.

IL-8 was secreted and expressed by mesenchymal cell lines, whereas the proneural cell line did not express *CXCL8* and displayed minimal IL-8 secretion. These findings confirm previous association of IL-8 with mesenchymal glioma stem-like cells (GSCs).^28^ IL-8 has also been suggested to recruit neutrophils in glioma supporting our finding of *CXCL8* association with neutrophils.^29^ The dual association we found of *CXCL8* with NK cells is less clear. However, special subsets of NK cells increase IL-8 secretion from GSCs^30^ implying complex TME crosstalk involving IL-8. Although our results do not exclude that tumor IL-8 production may contribute to plasma IL-8, it is hardly the sole source. Patients with astrocytomas have increased numbers of IL-8 secreting peripheral blood mononuclear cells compared to healthy controls^31^ and *CXCL8* expression in glioma tissue was not associated with OS in the TCGA cohort, supporting the hypothesis that plasma IL-8 has independent prognostic potential. In addition, IL-8 is a cytokine elevated in plasma of patients suffering from a number of infectious diseases and conditions characterized by inflammation.^32,33^ High plasma IL-8 could therefore also reflect presence of systemic inflammation in these patients.

Soluble receptors and ligands are produced by mRNA expression, posttranscriptional regulation, or cleavage of membrane-bound proteins.^34,35^ These soluble variants may interact with full-length ligands and receptors and thereby regulate immune responses.^34^ Among the proteins we found associated with diagnosis and/or prognosis several were soluble immune checkpoint proteins. Plasma soluble ICOSLG was the only checkpoint molecule associated with OS in multivariate analysis. A recent study found that ICOSLG was expressed and secreted by mesenchymal GBM cells^36^ and absent in normal brain,^36,37^ and this is similar to our findings. Iwata et al. suggest that soluble ICOSLG released from mesenchymal GBM cells induces CD4+ICOS+Foxp3+T cells (regulatory T cells), IL-10 production and suppression of general T cell proliferation. They also found that ICOSLG expression in GBM is inversely correlated with patient survival and induce tumorigenicity in a preclinical model.^36^ These results indicate that soluble ICOSLG in the TME may suppress antitumor immune responses through regulatory T cells and IL-10. In myasthenia gravis high soluble ICOSLG is suggested to induce peripheral blood follicular helper T (Tfh) cell activation and proliferation.^38^ Tfh cell levels in glioma tissue have been found to be negatively related to prognosis^39^ and a predictor for malignant transformation from low-grade to high-grade gliomas.^40^ Paradoxically, we found high plasma ICOSLG to be associated with long OS in newly diagnosed GBM; *ICOSLG* expression was not associated with survival in the TCGA GBM dataset; and immune cell deconvolution of TCGA GBM samples revealed that *ICOSLG* expression was not correlated with regulatory T cells and negatively correlated with the presence of Tfh cells, adding to the complexity of ICOSLG immune regulation. Furthermore, ICOSLG has also been shown to induce CD8+ cytotoxic lymphocyte-mediated antitumor response^41,42^ and induces both Th1 and Th2 cytokine production in a glioma model.^37^ Reasons for these discrepancies could be: (1) dual and/or tissue specific functions of ICOSLG; (2) secretion of soluble ICOSLG from nontumor cells impacting antitumor immunity; (3) crosstalk between cells in the TME affecting ICOSLG function; and (4) independent functions of membrane-bound, soluble and circulating ICOSLG.

Our data support, that ICOSLG may have diverse roles in immune regulation in GBM. Nevertheless, immune cell fractions in gliomas are low^43^ and cells expected to contribute to antitumor immunity may be inactivated or even support immune evasion in the brain TME.^44,45^

For all 8 cell lines examined ICOSLG values were higher in plasma than in conditioned medium from paired GBM cell lines. In addition, in silico analysis revealed limited *ICOSLG* expression in the brain TME, suggesting that plasma ICOSLG is produced outside the TME and may have independent functions indirectly affecting GBM prognosis.

Limitations of our study include possible impact of surgery (perioperative blood sampling) and corticosteroid treatment on plasma protein levels; incomplete molecular characterization of tumor samples; lack of serial sampling, healthy controls, and independent validation. Only 2 patients did not receive corticosteroid therapy and there was no association between corticosteroid dosage and plasma IL-8 or ICOSLG in the 82 patients with newly diagnosed GBM and known corticosteroid consumption at surgery, making it less likely that corticosteroid use is an important confounder in this setting. However, it is plausible that corticosteroid use may have been associated with plasma levels of other candidates in the panel of 92 proteins, and therefore obscured their association with survival.

Multivariate analysis was performed with stepwise selection with 10-fold cross-validation and a selection criterion of a 1% *P* in order to minimize the risk of overfitting. No correction for multiple testing was done in the initial selection of candidate markers as it was considered unnecessary. Nor was a correction for multiple testing done for the final multivariate model as this is an exploratory study.^46^

Prospective studies as well as studies on patients treated with immune therapy are warranted to test applicability of these potential biomarkers. Described limitations should be addressed in future studies, since they may have major impact on results and conclusions concerning immune-related blood-based biomarkers.

Although recent years’ research has cemented the important role of the brain TME and local immune response in brain tumor growth,^44,47^ our results suggest that some circulating immune modulators may impact GBM propagation differently than when present in the brain TME. This highlights complexity of GBM immune modulation and calls for caution when selecting targets for immunotherapy.

## Funding

University of Copenhagen [Faculty Scholarship to C.B.H.]; Læge Sofus Carl Emil Friis og hustru Olga Doris Friis’ Legat; Danish Cancer Society [R146-A9511 to P.H., R148-A10151 to P.H.].

## Supplementary Material

vdab072_suppl_Supplementary_Materials
