# Supplementary material for: Plasma IL-8 and ICOSLG as prognostic biomarkers in glioblastoma
Source: Neurooncol Adv. 2021 Jun 1;3(1):vdab072. doi: 10.1093/noajnl/vdab072 (PMC8284624; doi:10.1093/noajnl/vdab072)
Supplement: vdab072_suppl_Supplementary_Materials [file vdab072_suppl_supplementary_materials.pdf]

Plasma IL-8 and ICOSLG as prognostic biomarkers in glioblastoma

Supplementary Data

Table S1. Immune-related proteins used for analysis, including LOD and percentage of missing data.

Assay	Uniprot ID	OlinkID	LOD	Missing data freq.
	**Olink NPX Manager 0.0.104.0**	**Olink IMMUNO-ONCOLOGY(v.3101)**		
ADA	P00813	OID00775	-0,06858	0%
ADGRG1	Q9Y653	OID00764	1,09853	66%
ANG-1	Q15389	OID00760	0,96913	0%
ANGPT2	O15123	OID00822	0,21105	0%
ARG1	P05089	OID00815	2,91545	34%
CAIX	Q16790	OID00773	0,76390	0%
CASP-8	Q14790	OID00827	0,90698	0%
CCL17	Q92583	OID00821	0,48503	0%
CCL19	Q99731	OID00794	1,43011	0%
CCL20	P78556	OID00837	1,41494	0%
CCL23	P55773	OID00811	0,48554	0%
CCL3	P10147	OID00813	0,48405	0%
CCL4	P13236	OID00796	0,76242	0%
CD244	Q9BZW8	OID00758	1,70355	0%
CD27	P26842	OID00800	0,76846	0%
CD28	P10747	OID00793	0,79042	84%
CD4	P01730	OID00776	-1,20711	0%
CD40	P25942	OID00781	0,99210	0%
CD40-L	P29965	OID00756	1,82765	0%
CD5	P06127	OID00812	-0,35696	0%
CD70	P32970	OID00808	1,10837	0%
CD83	Q01151	OID00841	0,40025	0%
CD8A	P01732	OID00772	1,02123	0%
CRTAM	O95727	OID00766	1,41125	0%
CSF-1	P09603	OID00843	1,05081	0%
CX3CL1	P78423	OID00806	1,02620	0%
CXCL1	P09341	OID00786	1,08105	0%
CXCL10	P02778	OID00807	0,76898	0%
CXCL11	O14625	OID00767	1,09658	0%
CXCL12	P48061	OID00824	1,09180	60%
CXCL13	O43927	OID00830	1,73236	0%
CXCL5	P42830	OID00801	2,07689	0%
CXCL9	Q07325	OID00771	0,96783	0%
DCN	P07585	OID00817	0,77150	0%
EGF	P01133	OID00759	0,90500	0%
FASLG	P48023	OID00792	0,40674	0%

FGF2	P09038	OID00770	0,07186	55%
Gal-1	P09382	OID00798	1,54188	0%
Gal-9	O00182	OID00779	0,57521	0%
GZMA	P12544	OID00804	0,74031	0%
GZMB	P10144	OID00840	0,80241	0%
GZMH	P20718	OID00783	1,81638	1%
HGF	P14210	OID00803	0,90257	0%
HO-1	P09601	OID00805	2,49527	0%
ICOSLG	O75144	OID00828	0,43372	0%
IFN-beta	P01574	OID00774	0,47683	93%
IFN-gamma	P01579	OID00825	0,49844	98%
IL-1 alpha	P01583	OID00757	1,02999	98%
IL10	P22301	OID00809	1,71873	7%
IL12	P29459,P29460	OID00842	2,08708	7%
IL12RB1	P42701	OID00835	1,09594	33%
IL13	P35225	OID00836	1,03854	92%
IL18	Q14116	OID00782	1,33730	0%
IL2	P60568	OID00778	0,48512	95%
IL-21	Q9HBE4	OID00834	1,10731	99%
IL33	O95760	OID00788	1,22384	98%
IL-35	Q14213,P29459	OID00797	1,76262	99%
IL4	P05112	OID00833	0,23474	77%
IL5	P05113	OID00802	1,97464	81%
IL6	P05231	OID00763	1,25271	3%
IL7	P13232	OID00761	0,92327	0%
IL8	P10145	OID00752	2,46291	0%
KLRD1	Q13241	OID00839	0,79048	0%
LAMP3	Q9UQV4	OID00826	1,39679	0%
LAP TGF-beta-1	P01137	OID00785	-0,47638	0%
MCP-1	P13500	OID00765	0,81881	0%
MCP-2	P80075	OID00795	1,02885	0%
MCP-3	P80098	OID00755	1,29232	84%
MCP-4	Q99616	OID00768	0,25783	0%
MIC-A/B	Q29983,Q29980	OID00820	0,53844	8%
MMP12	P39900	OID00829	0,95760	0%
MMP7	P09237	OID00814	1,98314	0%
NCR1	O76036	OID00816	1,38045	0%
NOS3	P29474	OID00777	1,50517	68%
PDCD1	Q15116	OID00791	1,70208	29%
PDGF subunit B	P01127	OID00790	1,07446	0%
PD-L1	Q9NZQ7	OID00799	2,50123	0%
PD-L2	Q9BQ51	OID00831	0,44794	0%
PGF	P49763	OID00762	1,20723	0%
PTN	P21246	OID00823	0,69149	27%

TIE2	Q02763	OID00754	0,84503	0%
TNF	P01375	OID00838	1,45289	94%
TNFRSF12A	Q9NP84	OID00810	0,85042	0%
TNFRSF21	O75509	OID00818	0,29462	0%
TNFRSF4	P43489	OID00819	1,60074	3%
TNFRSF9	Q07011	OID00753	0,04428	0%
TNFSF14	O43557	OID00787	0,41363	0%
TRAIL	P50591	OID00769	0,04635	0%
TWEAK	O43508	OID00789	-0,33856	0%
VEGFA	P15692	OID00832	0,39146	0%
VEGFC	P49767	OID00784	-0,09811	38%
VEGFR-2	P35968	OID00780	0,32967	0%

OLINK Immuno-Oncology panel v. 3101. Missing data freq: Percentage of data below LOD or missing. Proteins in red have >10% missing data. Abbreviations: LOD, limit of detection.

Table S2. Patient characteristics for Cohort 1 (Gliomas WHO grade II-IV).

**Characteristics (Cohort 1)**	**Gliomas WHO grade II-IV (n=158)**
**Age, years median (range)**	60 (23-89)
**Sex, n (%)**	
Male	94 (59.5)
Female	64 (40.5)
**Diagnosis, n (%)**	**n=158**
Oligodendroglioma WHO grade II-III	7 (4.4)
Astrocytoma WHO grade II-III	17 (10.8)
GBM	134 (84.8)
**Survival outcome**	**n=156**
OS, weeks median (range)	58 (1-233)
Lost to follow-up	2
**Status at end of follow-up, n (%)**	**n=156**
Dead	116 (74.4)
Alive	40 (25.6)
Lost to follow-up	2

Abbreviation: OS: overall survival.

Table S3. Patient characteristics for Cohort 2 (Newly diagnosed GBM).

**Characteristics (Cohort 2)**	**Newly diagnosed GBM (n=94)**
**Age, years median (range)**	65 (33-89)
**Sex, n (%)**	
Male	57 (60.6)
Female	37 (39.4)
**Treatment, n (%)**	**n=94**
No treatment	7 (7.4)
Radiation therapy only	3 (3.2)
Temozolomide (TMZ) only	2 (2.1)
Stupps Regimen	65 (69.1)
Radiation therapy and concomittant TMZ	2 (2.1)
Palliative radiation therapy	15 (16)
Missing information	0
**Steroid treatment at blood sampling, n (%)**	**n=82**
0	2 (2.4)
12.5-25 mg	3 (3.7)
50-75 mg	28 (34.1)
87.5-100 mg	46 (56.1)
>100 mg	3 (3.7)
Missing information	12
**MGMT promotor methylation status, n (%)**	**n=91**
Methylated	40 (44)
Non-methylated	51 (56)
Missing information	3
**Survival outcome**	
PFS, weeks median (range)	23 (3-233)
Missing (Uncertainty regarding recurrence date), n	1
OS, weeks median (range)	58 (3-233)
Lost to follow-up, n	2

Abbreviations: OS: overall survival; PFS: progression-free survival.

Table S4. Patient characteristics for Cohort 3 (Recurrent GBM).

**Characteristics (Cohort 3)**	**Recurrent GBM (n=40)**
**Age, years median (range)**	56 (37-79)
**Sex, n (%)**	
Male	22 (55)
Female	18 (45)
**Primary treatment regimen, n= 38 (%)**	**n=38**
No treatment	1 (2.6)
Radiation therapy only	0
Temozolomide (TMZ) only	0
Stupps Regimen	35 (92.1)
Radiation therapy and concomittant TMZ	0
Palliative radiation therapy	2 (5.3)
Missing information	2
**Number of recurrences, n (%)**	**n=39**
First recurrence GBM	25 (64.1)
Second recurrence GBM	12 (30.8)
Third recurrence GBM	2 (5.1)
Missing information	1
**Treatment, n (%)**	**n=38**
No treatment	18 (47.4)
Radiation therapy only	1 (2.6)
Temozolomide only	6 (15.8)
Lomustine only	1 (2.6)
Avastin and Lomustine	5 (13.2)
Clinical trial	5 (13.2)
Radiation therapy and Temozolomide	2 (5.2)
Missing information	2
**Survival outcome**	
OS (from blood sampling to death or end of follow-up), weeks median (range)	33 (2-102)
OS (from diagnosis to death or end of follow-up), weeks median (range)	97 (30-785)

Abbreviation: OS: overall survival.

Table S5. Immune-related proteins in plasma associated with prognosis in newly diagnosed GBM – Univariate analysis.

**Univariate analysis**	**PFS**		**OS**	
**Protein**	**HR (95% CI)**	***P***	**HR (95% CI)**	***P***
Adenosine deaminase (ADA)	0.48 (0.28-0.81)	**0.0060**	0.44 (0.25-0.79)	**0.0063**
Arginase-1 (ARG1)	0.74 (0.53-1.02)	0.062	0.69 (0.49-0.97)	**0.035**
Caspase-8 (CASP_8)	0.72 (0.53-0.98)	**0.039**	0.76 (0.56-1.03)	0.078
T-cell surface glycoprotein CD4 (CD4)	0.49 (0.22-1.08)	0.076	0.39 (0.17-0.92)	**0.031**
T-cell surface glycoprotein CD5 (CD5)	0.58 (0.35-0.97)	**0.037**	0.55 (0.31-0.97)	**0.039**
T-cell-specific surface glycoprotein CD28 (CD28)	0.34 (0.14-0.84)	**0.018**	0.44 (0.18-1.08)	0.074
Natural killer receptor 2B4 (CD244)	0.33 (0.19-0.58)	**<.0001**	0.35 (0.19-0.62)	**0.0004**
C-X-C motif chemokine 5 (CXCL5)	0.84 (0.72-0.98)	**0.029**	0.81 (0.69-0.95)	**0.012**
Granzyme B (GZMB)	0.83 (0.65-1.06)	0.14	0.77 (0.60-0.99)	**0.042**
Galectin-1 (Gal-1)	0.41 (0.20-0.86)	**0.018**	0.51 (0.23-1.12)	0.094
ICOS ligand (ICOSLG)	0.29 (0.13-0.63)	**0.0017**	0.21 (0.09-0.51)	**0.0005**
Interleukin-8 (IL-8)	1.56 (1.18-2.07)	**0.0019**	1.41 (1.06-1.88)	**0.019**
Natural cytotoxicity triggering receptor (NCR1)	0.51 (0.28-0.92)	**0.025**	0.65 (0.35-1.21)	0.17
Programmed cell death protein 1 (PDCD1)	0.60 (0.37-0.97)	**0.035**	0.57 (0.33-0.97)	**0.039**
Programmed cell death 1 ligand 1 (PD-L1)	0.62 (0.39-1.00)	**0.048**	0.61 (0.37-1.01)	0.054
Pleiotrophin (PTN)	1.20 (1.00-1.45)	0.050	1.24 (1.03-1.49)	**0.026**
Angiopoietin-1 receptor (TIE2)	0.44 (0.20-0.99)	**0.047**	0.41 (0.18-0.96)	**0.040**
TNF-related apoptosis-inducing ligand (TRAIL)	0.39 (0.21-0.75)	**0.0045**	0.50 (0.24-1.01)	0.054
Tumor necrosis factor ligand superfamily member 12 (TWEAK)	0.40 (0.19-0.83)	**0.014**	0.42 (0.19-0.93)	**0.032**
Vascular endothelial growth factor receptor 2 (VEGFR-2)	0.51 (0.27-0.97)	**0.039**	0.45 (0.22-0.93)	**0.030**

Abbreviations: CI, confidence interval; GBM, glioblastoma; HR, hazard ratio; OS, overall survival; PFS, progression-free survival.

Table S6. Correlation between *CXCL8*, *ICOSLG* and immune cell fractions in TCGA dataset.

Gene name	Cell type	Correlation	*P*
ICOSLG	B cells memory	0,02762681	0,605449487
ICOSLG	B cells naive	-0,08376948	0,11668919
ICOSLG	Dendritic cells activated	-0,284980577	5,30046E-08
ICOSLG	Dendritic cells resting	0,052054803	0,330145324
ICOSLG	Eosinophils	-0,030688083	0,56607154
ICOSLG	Macrophages M0	0,094372037	0,077023009
ICOSLG	Macrophages M1	0,055560329	0,298572484
ICOSLG	Macrophages M2	0,120377382	0,023904181
ICOSLG	Mast cells activated	0,020831566	0,696915988
ICOSLG	Mast cells resting	-0,133158318	0,01240076
ICOSLG	Monocytes	0,0821432	0,123984602
ICOSLG	Neutrophils	0,192539451	0,000279531
ICOSLG	NK cells activated	-0,226022761	1,86051E-05
ICOSLG	NK cells resting	0,193494777	0,000260264
ICOSLG	Plasma cells	-0,008695561	0,87086019
ICOSLG	T cells CD4 memory activated	0,153472894	0,003898215
ICOSLG	T cells CD4 memory resting	-0,188904367	0,000365676
ICOSLG	T cells CD4 naive	-0,037680315	0,481008716
ICOSLG	T cells CD8	0,046201591	0,387479669
ICOSLG	T cells follicular helper	-0,416442139	3,37932E-16
ICOSLG	T cells gamma delta	-0,004384421	0,934673095
ICOSLG	T cells regulatory (Tregs)	-0,007416498	0,889724512
CXCL8	B cells memory	0,004154324	0,938094432
CXCL8	B cells naive	-0,076726124	0,150857923
CXCL8	Dendritic cells activated	-0,092402266	0,083423387
CXCL8	Dendritic cells resting	0,037823329	0,479344286
CXCL8	Eosinophils	0,004511816	0,932779351
CXCL8	Macrophages M0	0,140126349	0,008472078
CXCL8	Macrophages M1	-0,217077881	4,00247E-05
CXCL8	Macrophages M2	-0,153158447	0,003972988
CXCL8	Mast cells activated	0,305518663	4,85225E-09
CXCL8	Mast cells resting	-0,315620351	1,39559E-09
CXCL8	Monocytes	0,008495257	0,873809837
CXCL8	Neutrophils	0,398185194	7,99864E-15
CXCL8	NK cells activated	-0,301383819	7,97268E-09
CXCL8	NK cells resting	0,301781121	7,60377E-09
CXCL8	Plasma cells	-0,009128922	0,864484748
CXCL8	T cells CD4 memory activated	0,108616395	0,041689669
CXCL8	T cells CD4 memory resting	-0,134916211	0,011281894
CXCL8	T cells CD4 naive	0,021191795	0,691940083
CXCL8	T cells CD8	-0,030051734	0,574155565
CXCL8	T cells follicular helper	-0,318219319	1,00507E-09
CXCL8	T cells gamma delta	-0,068903308	0,197163298
CXCL8	T cells regulatory (Tregs)	0,075696342	0,156432739

Immune cell fractions used were estimated by Wang and colleagues[1]. Abbreviations: ICOSLG, ICOS ligand.

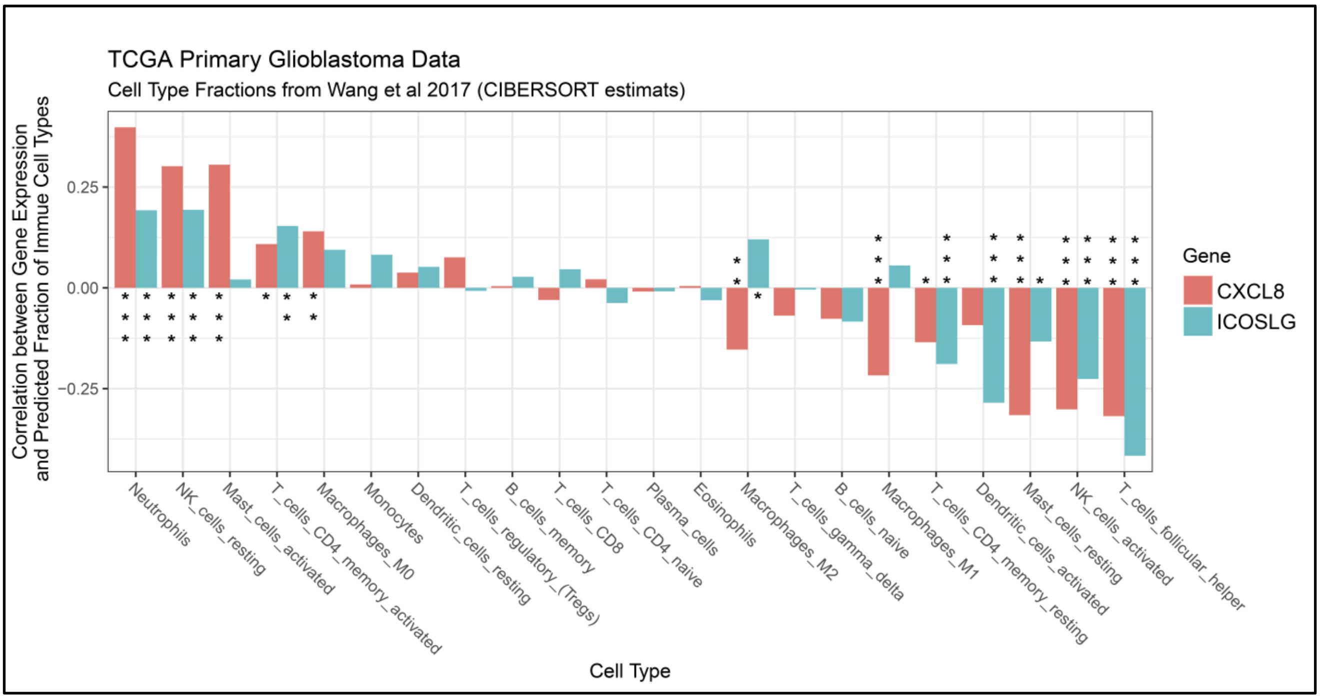

Figure S1. Correlation between *CXCL8*, *ICOSLG* and immune cell fractions in TCGA dataset. Immune cell fractions used were estimated by Wang and colleagues[1]. Abbreviations: ICOSLG, ICOS ligand. Statistical significance: **P*<0.05; ***P*<0.01; ****P*<0.001.

Supplement reference

1. Wang Q, Hu B, Hu X, et al. Tumor evolution of glioma-intrinsic gene expression subtypes associates with immunological changes in the microenvironment. Cancer cell. 2017; 32(1):42-56.e46.
